# Blood phosphorylated Tau217 distinguishes amyloid-positive from amyloid-negative subjects in the Alzheimer's disease continuum. A systematic review and meta-analysis

**DOI:** 10.1007/s00415-025-12996-3

**Published:** 2025-03-06

**Authors:** Annibale Antonioni, Emanuela Maria Raho, Francesco Di Lorenzo, Lamberto Manzoli, Maria Elena Flacco, Giacomo Koch

**Affiliations:** 1 Doctoral Program in Translational Neurosciences and Neurotechnologies, Department of Neuroscience and Rehabilitation, University of Ferrara, Via Ludovico Ariosto, 35, 44121 Ferrara, Italy; 2 Department of Neuroscience and Rehabilitation, University of Ferrara, 44121 Ferrara, Italy; 3 Department of Neuroscience, Ferrara University Hospital, 44124 Ferrara, Italy; 4 Neuropsychophysiology Lab, Santa Lucia Foundation IRCCS, Via Ardeatina, 306, 00179 Rome, Italy; 5 Department of Medical and Surgical Sciences, University of Bologna, 40126 Bologna, Italy; 6 Department of Environmental and Prevention Sciences, University of Ferrara, 44121 Ferrara, Italy; 7 Section of Physiology, Department of Neuroscience and Rehabilitation, University of Ferrara, 44121 Ferrara, Italy; 8 Center for Translational Neurophysiology, Istituto Italiano di Tecnologia, 44121 Ferrara, Italy

**Keywords:** Alzheimer's disease (AD), Biomarkers, Blood, Dementia, Mild cognitive impairment (MCI), Phosphorylated Tau 217 (pTau217)

## Abstract

**Background:**

Alzheimer’s disease (AD) is the leading cause of dementia worldwide, and cost-effective tools to detect amyloid pathology are urgently needed. Blood-based Tau phosphorylated at threonine 217 (pTau217) seems promising, but its reliability as a proxy for cerebrospinal fluid (CSF) status and ability to identify patients within the AD spectrum remain unclear.

**Methods:**

We performed a systematic review and meta-analysis on the potential of blood pTau217 to differentiate amyloid-positive (A+) and amyloid-negative (A−) subjects. We included original studies reporting quantitative data on pTau217 concentrations in both blood and CSF in the AD continuum. The single-group meta-analysis computed the pooled pTau217 levels in blood and in CSF, separately in the A+ and A− groups, while the head-to-head meta-analysis compared the mean pTau217 concentrations in the A+ versus A− subjects, both in blood and CSF, stratifying by assessment method in both cases.

**Results:**

Ten studies (819 A+; 1055 A−) were included. The mean pTau217 levels resulted higher in CSF than in blood and, crucially, in A+ individuals than in A– ones, regardless of the laboratory method employed. Most importantly, all laboratory techniques reliably distinguished A+ from A– subjects, whether applied to CSF or blood samples.

**Conclusions:**

These results confirm that blood-based pTau217 is a reliable marker of amyloid pathology with significant implications for clinical practice in the AD continuum. Indeed, pTau217 might be a non-invasive, scalable biomarker for early AD detection, reducing the reliance on more invasive, expansive, and less accessible methods.

**Clinical trial registration:**

Prospero CRD42024565187

**Supplementary Information:**

The online version contains supplementary material available at 10.1007/s00415-025-12996-3.

## Introduction

Alzheimer's disease (AD) is the most common neurodegenerative dementia worldwide, and the number of affected individuals is expected to rise dramatically in the coming decades [1]. Given its burden, current research is focused on identifying biomarkers that allow for its early identification, which is critical for addressing modifiable risk factors to slow disease progression and for enrolling patients eligible for novel disease-modifying therapies [2, 3]. Traditionally, the detection of AD neuropathological hallmarks, i.e., aggregated amyloid-β42 (Aβ42) and Tau phosphorylated at threonine 181 (pTau181), has relied on cerebrospinal fluid (CSF) analysis and nuclear medicine techniques such as positron emission tomography (PET) [4]. While these methods have been pivotal in diagnosing AD, they are invasive, expansive, not readily accessible in primary care settings, and require specialized expertise and time [5]. Thus, in recent years, extensive research has been devoted to exploring the diagnostic potential of blood-based biomarkers, which might offer a less invasive and more widely available alternative [6]. Indeed, since CSF pathological biomarkers cross the blood-brain barrier (BBB), they may also be detected in the blood [7]. As Aβ is one of the most characteristic AD neuropathological features, there were high expectations for its new blood measurement techniques [8]. However, blood Aβ assessment methods showed relevant limitations [9]. Thus, attention focused on the other blood AD-specific biomarker, i.e., pTau, which highlighted a considerable potential. Consistently, a recent review has suggested a correlation between CSF and blood pTau181 levels, with higher concentrations documented in AD patients compared to other neurodegenerative diseases [10]. Moreover, blood biomarkers also show promise in differentiating between healthy individuals, those with mild cognitive impairment (MCI), and AD patients [11, 12]. However, importantly, various isoforms of blood pTau exist, distinguished by their phosphorylation sites, and although pTau181 is currently the most extensively studied one with a well-established diagnostic role, other isoforms, such as pTau217, are emerging as highly promising [13]. Specifically, while pTau181 mainly correlates with amyloid deposits and neurodegeneration markers in the moderate to advanced stages of the disease, pTau217 appears to be more sensitive in the earliest stages, when amyloid and tau deposits are still limited [10, 14–16]. This suggests that blood pTau217 may allow the detection of the initial neuropathological changes associated with AD, even in clinically asymptomatic or mildly symptomatic individuals. However, to our knowledge, no systematic review has directly and quantitatively evaluated the relationship between blood and CSF pTau217 levels to assess whether blood measures can reliably reflect CSF status across the AD continuum. This is critical because most results come from individual studies, often with limited sample sizes and varying laboratory analysis methods, making it difficult to generalize findings [10]. Therefore, there remains inconsistency in the literature regarding the ability of this blood-based biomarker to describe the CSF status, i.e., the central nervous system (CNS) compartment where the AD neuropathological process occurs. Given that pTau217 appears to be an early AD biomarker, even in presymptomatic stages, demonstrating a reliable correlation between its blood and CSF levels could make blood-based pTau217 a pivotal tool in clinical and research settings [10, 14–16]. Indeed, it would not only enable the early identification of patients eligible for novel disease-modifying therapies, which are most effective when neuropathological damage is still minimal but also facilitate rapid, non-invasive monitoring of therapeutic responses during clinical trials [17, 18].

Here, we performed a systematic review of the literature on pTau217 concentrations in CSF and blood. Moreover, we conducted two meta-analyses: the single group meta-analysis computed the pooled pTau217 levels in blood and in CSF, separately in the Aβ-positive (A+) and Aβ-negative (A−) groups, while the head-to-head meta-analysis compared the mean pTau217 concentrations in the A+ versus A− subjects, both in blood and in CSF, stratifying by assessment method in both cases. We aim to provide clinicians and researchers with evidence on the reliability of blood pTau217 as a proxy for CSF status in identifying patients in the AD spectrum.

## Materials and methods

### Bibliographic search, data extraction, and quality assessment

This systematic review and meta-analysis followed the updated version of the Preferred Reporting Items for Systematic Reviews and Meta-Analysis (PRISMA) guidelines (Additional file 1) [19]. The study protocol was registered in the “International Prospective Register of Systematic Reviews” (PROSPERO, CRD42024565187). Specifically, the MEDLINE (via PubMed), Scopus (via EBSCO), and Web of Science databases were searched up to 31 July 2024 for studies reporting measurements of pTau217 on both blood and CSF in patients in the AD continuum, i.e., MCI and overt AD dementia patients categorised according to neuropathological and/or clinical criteria. The following search strategy was employed, without language restrictions: ((((pTau[Title/Abstract]) OR (pTau[Title/Abstract])) AND ((cerebrospinal fluid[Title/Abstract]) OR (CSF[Title/Abstract])) AND (blood[Title/Abstract])) AND (Alzheimer[Title/Abstract])) The reference lists of retrieved paper and reviews were also screened for additional suitable papers. Inclusion criteria were: (1) case series or cross-sectional design, either published as primary analyses or as sub-analyses of larger population-based cohort studies; (2) original quantitative data about pTau217 values on blood and CSF in patients on the AD continuum. When possible, the same data were also collected on comparators, e.g. (a) subsets of subjects classified according to different degrees of disease severity (e.g. MCI vs. AD); (b) clinically healthy controls; (c) biomarker-negative subjects; (d) different types of dementia (e.g. Lewy-Body dementia, frontotemporal dementia). Preclinical studies (e.g., on cellular and animal models) and different publication types (e.g., review, pre-print, commentary) were excluded. Each included article was independently evaluated by two reviewers (AA and EMR) who extracted the study characteristics and measures of interest. In case of a lack of agreement on the work to be included or discrepancies in data extraction, a third author was contacted (FDL) to achieve consensus through discussion. In particular, in the first phase, titles and abstracts were blinded against inclusion and exclusion criteria. In the subsequent phase, the full text of the articles selected in the previous stage was obtained, and the eligibility criteria were reapplied. Individual study quality was assessed using an adapted version of the Newcastle Ottawa Quality Assessment Scale to evaluate the comparability across groups for confounding factors, the appropriateness of outcome assessment, length of follow-up, and missing data handling and reporting [20]. The data extracted included sample size, diagnostic category(ies) (according to clinical and/or neuropathological criteria, where available), pTau217 measurements on both blood and CSF in AD continuum patients, any comparator(s), and laboratory technique(s) used. If the data could not be extracted from the selected reports, the authors were contacted three times within 3 weeks to request the necessary data.

## Data analysis

In the first phase, data from individual studies were combined to estimate the weighted mean (WM) concentration (plus 95% confidence intervals, CIs) of pTau217 in the CSF and blood of A+ and A− subjects, using the metan package in Stata. When multiple values of pTau217 were available for the same study (e.g., evaluated by AD subgroups or age class), a summary WM was recomputed within the same population. Each analysis was performed separately by the assessment method. Subsequently, we performed traditional head-to-head meta-analyses, combining individual study pTau217 levels of A+ and A− subjects, considering each time: (1) CSF levels, (2) blood concentration, and (3) assessment method. We employed the random-effect model and computed a summary mean difference, its 95% CI, and the relative intrA−study heterogeneity (quantified using the I^2^ metric). For each outcome, the total number of publications included in the meta-analyses was <10, thus we were not able to assess publication bias, either graphically, through funnel plots, or formally, through Egger's regression asymmetry test (in such cases, the power is too low to distinguish chance from real asymmetry) [21]. RevMan 5.3 (The Cochrane Collaboration, 2014) and Stata, version 13.1 (Stata Corp. College Station, TX: 2013) were used to analyze the data.

## Results

### Study selection and characteristics

**Fig. 1:**
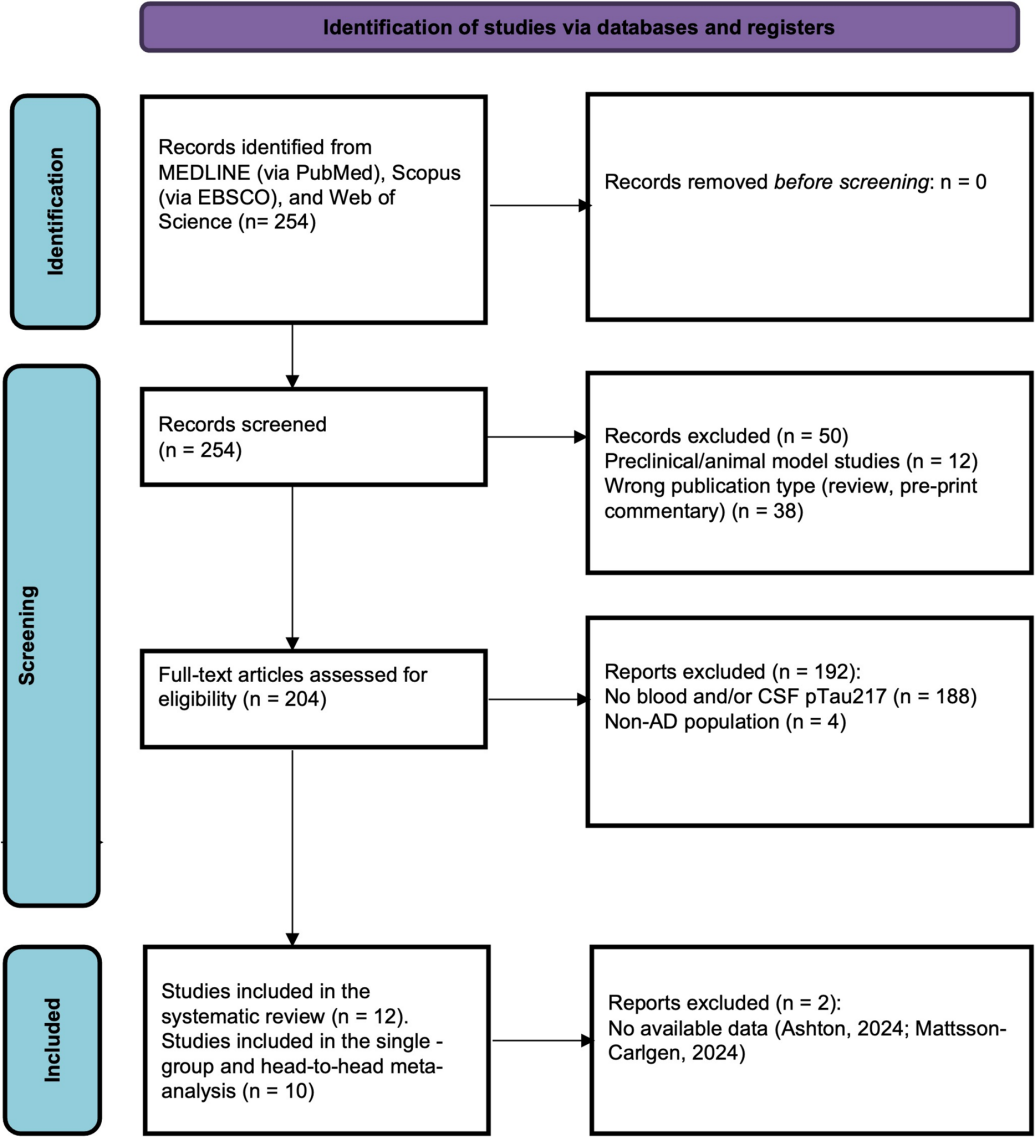
PRISMA flow diagram outlining literature review and study selection

Of the 254 records initially identified through the database search, 50 were immediately excluded for reasons related to the study population (preclinical or animal model studies, *n* = 12) or publication type (review, pre-print, commentary, *n* = 38). Thus, 204 articles were screened, and among these, 192 were excluded as they did not focus on the levels of pTau217 in blood and/or CSF (*n* = 188) or did not deal with the AD patients population (*n* = 4). Therefore, 12 studies were included in the systematic review [14, 15, 22–31]. However, two studies were excluded because it was not possible to obtain the necessary data [14, 26]. Consequently, ten studies were included in the single group and head-to-head meta-analysis. Figure 1 shows an overview of the research process, while Table 1 outlines the key data extracted from each study (see Fig. 1 and Table 1).

**Table 1:** Selected characteristics of the included studies

First author (Ref)	Year	Mean age (SD)	% males	Assessment method	N. A+ subjects	Mean CSF A+	SD CSF A+	Mean blood A+	SD blood A+	N. A− subjects	Mean CSF A−	SD CSF A−	Mean blood A−	SD blood A−
Ashton (a) [22]	2023	72.3 (5.8)	44.7	Janssen (Simoa)	127 (AD CSF profile)	18.17	11.3–30.1	0.12	0.07–0.201	70 (non AD CSF profile)	2.13	0.8–3.41	0.023	0.014–0.039
Ashton (b) [22]	2023	72.3 (5.8)	44.7	Lilly (MSD)	127 (AD CSF profile)	32.3	20.3–44.9	0.49	0.36–0.74	70 (non AD CSF profile)	4.5	2.85–7.34	0.15	0.12–0.20
Janelidze (a) [15]	2023	78.0 (73.5–81)	66	WashU	45 (MCI-ADD); 26 non progressors A+	11.6; 8.5	9.9–13.4; 5.4–9	3.49; 1.88	2.91–4.73; 1.27–2.73	64 non progressors A−	2.9	2.4–3.3	0.753	0.614–0.951
Janelidze (b) [15]	2023	78.0 (73.5–81)	66	Lilly (MSD)	45 (MCI-ADD); 26 non progressors A+	29.3; 12.6	17.9–43.3; 8–18.6	0.442; 0.275	0.33–0.532; 0.2–0.359	64 non progressors A−	5	2.7–6.7	0.177	0.146–0.201
Janelidze (c) [15]	2023	78.0 (73.5–81)	66	Janssen (Simoa)	45 (MCI-ADD); 26 non progressors A+	19.7; 9.7	13.6–26.1; 4.5–12.6	0.109; 0.066	0.077–0.173; 0.036–0.104	64 non progressors A−	3.6	1.6–4.3	0.034	0.020–0.049
Groot (a) [25]	2022	71.6 (5.8)	40	Janssen (Simoa)	45 (MCI-AD A+); 9 (MCI-other A+); 18 (Stable MCI A+)	25.29; 6.61; 12.27	23.38; 3.52; 7.22	0.13; 0.09; 0.07	0.08; 0.03; 0.04	24 (MCI-other A−); 51 (Stable MCI A−)	4.72; 3.58	3.75; 2.38	0.05; 0.04	0.04; 0.07
Groot (b) [25]	2022	71.6 (5.8)	40	Lilly (MSD)	45 (MCI-AD A+); 9 (MCI-other A+); 18 (Stable MCI A+)	38.28; 10.02; 19.8	30.62; 4.49; 14.6	0.46; 0.30; 0.31	0.18; 0.15; 0.11	24 (MCI-other A−); 51 (Stable MCI A−)	8.03; 5.09	6.96; 3.26	0.23; 0.20	0.11; 0.12
Barthélemy (a) [24]	2020	75 (5.0) (preclinical AD); 81 (7.0) (AD-MCI); 75 (2.0) (AD moderate)	80.0 (preclinical AD); 37.5 (AD-MCI); 0 (AD moderate)	Immunoprecipitation mass spectrometry (original method)	5 (preclinical AD); 8 (AD-MCI); 2 (AD moderate)	187; 247; 395	42; 58; 121	0.52; 0.82; 1.57	0.17; 0.52; 0.7	9 (young CN); 8 (aged CN); 2 (non AD-MCI)	54; 59; 56	5; 5; 20	0.13; 0.15; 0.13	0.02; 0.04; 0.01
Barthélemy (b) [24]	2020	74 (6.0) (preclinical AD); 76 (6.0) (AD-MCI); 74 (8.0) (AD moderate)	50.0 (preclinical AD); 54.2 (AD-MCI); 83.0 (AD moderate)	Immunoprecipitation mass spectrometry (original method)	20 (preclinical AD); 24 (AD-MCI); 6 (AD moderate)	184; 248; 355	159; 117; 206	0.26; 0.31; 0.58	0.25; 0.19; 0.5	31 (aged CN); 11 (non AD-MCI)	44; 43	16; 16	0.07; 0.09	0.03; 0.02
Palmqvist [29]	2019	72.6 (5.0)	56	Lilly (MSD)	151 A+	30	15.1	3.4	3.2	226 (A−)	17.9	6.7	2.1	5.3
Therriault [31]	2023	68.7 (7.8)	47.9	Quanterix (Simoa)	64 A+	24.15	18.25	0.1715	0.1383	110 A−	5.69	9.39	0.0487	0.0325
Mendes [27]	2024	72.9 (6.4) MCI; 71.1 (8.8) CI	51.0 (MCI); 50.0 (CI)	Lilly (MSD)	81	24.29; 44.31	23.49; 31.9	0.5; 0.7	0.42; 0.41	33 CU	13.42	9.96	0.25	0.3
Orduna Dolado [28]	2024	78 (4.4)	71.0	Lilly (MSD)	18	25.02 (morning); 27.00 (evening)	24.74; 28.98	0.47 (morning); 0.44 (evening)	0.16;0.16	20	5.03; 5.19	2.41; 2.68	0.29; 0.29	0.11;0.11
Bali [23]	2024	77.5 (74.8–80)	34.0	Lilly (MSD)	50	21.7	34.84–13.93	0.27	0.35–0.20	50	5.73	7.91–4.42	0.16	0.19–0.13
Therriault (a) [30]	2024	73.1 (CU A+); 71.0 (MCI+); 66.7 (AD)	8.2 (CU A+); 5.7 (MCI+); 7.8 (AD)	ALZpath (Simoa)	121	28.2; 45.6; 67.8	24.1–50.1; 29.0–77.7; 48.3–102	0.48; 0.79; 1.35	0.30–0.91; 0.53–1.11; 0.97–1.86	25 (young); 107 (CU-); 19 (MCI-); 22 (non AD)	6.69; 10.4; 11.4; 9.62	5.33–7.80; 7.37–16.1; 8.97–17.4; 6.56–11.1	0.16; 0.23; 0.31; 0.21	0.11–0.20; 0.16–0.32; 0.19–0.41; 0.16–0.35
Therriault (b) [30]	2024	73.1 (CU A+); 71.0 (MCI+); 66.7 (AD)	8.2 (CU A+); 5.7 (MCI+); 7.8 (AD)	Janssen (Simoa)	121	28.2; 45.6; 67.8	24.1–50.1; 29.0–77.7; 48.3–102	0.08; 0.13; 0.23	0.05–0.11; 0.10–0.18; 0.17–0.35	173	6.69; 10.4; 11.4; 9.62	5.33–7.80; 7.37–16.1; 8.97–17.4; 6.56–11.1	0.04; 0.04; 0.05; 0.05	0.02–0.04; 0.03–0.05; 0.04–0.07; 0.04–0.07

CSF and blood pTau217 levels expressed as pg/mL

*A+* Amyloid-positive; *A−* Amyloid-negative; *AD* Alzheimer’s disease; *ADD* Alzheimer’s disease Dementia; *CN* cognitively normal; *CSF* cerebrospinal fluid; *CU* cognitively unimpaired; *MCI* mild cognitive impairment; *MSD* Meso Scale Discovery; *N.* Number of subjects; *SD* standard deviation; *Simoa* Single-molecule array for protein detection

Overall, we included 819 participants classified as A+ and 1055 as A−, based on biomarkers [23, 28, 31] or biomarkers in combination with clinical diagnostic criteria [15, 22, 24, 25, 27, 29, 30]. Regarding laboratory methods used to assess pTau levels, the Meso Scale Discovery (MSD) platform was the most widely used [15, 22, 23, 25, 27–29], closely followed by the Single Molecule Array for Protein Detection (Simoa) [15, 22, 25, 30, 31]. Interestingly, two studies employed a novel approach, implementing a mass spectrometry assay technique either alone (namely, WashU) [15] or in combination with immunoprecipitation (IP-MS) [24], respectively. The methodological details of the included cohort and cross-sectional studies are outlined in Table 2 (see Tables 2 and 3). In brief, for the cohort studies, patient selection, outcome(s) ascertainment, and the assessment of subject comparability were optimal or adequate across nearly all investigations. Similarly, among the included cross-sectional studies, the same items proved optimal or adequate in all cases.

**Table 2:** The methodological quality of the included cohort studies according to the Newcastle-Ottawa Quality Assessment Scale

	Selection	Comparability	Outcome
	(max. score 4)	(max. score 2)	(max. score 3)
Groot, 2022 [25]	4	1	3
Orduña Dolado, 2024 [28]	3	1	2
Bali, 2024 [23]	3	1	3
Janelidze 2023 [15]	3	2	3

**Table 3:** The methodological quality of the included cross-sectional studies according to the Adapted Newcastle-Ottawa Quality Assessment Scale

	Selection	Comparability	Outcome
	(max. score 3)	(max. score 2)	(max. score 3)
Barthelemy 2020 [24]	1	1	3
Therriault, 2023 [31]	2	1	3
Mendes, 2024 [27]	3	1	3
Palmqvist, 2019 [29]	3	1	3
Therriault, 2024 [30]	3	1	3
Ashton 2023 [22]	1	0	3

### Single group meta-analysis

Table 4 provides an overview of the results of the ten studies included in the single-group meta-analysis (see Table 4).

**Table 4:** Cerebrospinal fluid (CSF) and blood pTau217 levels in Amyloid-positive (A+) and Amyloid-negative (A−) patients, stratified by assessment method

	A+ subjects			A− subjects		
		Cerebrospinal fluid*	Plasma*		Cerebrospinal fluid*	Plasma*
*Assessment method:*	N studies (sample)	Weighted mean (95% CI)	Weighted mean (95% CI)	N studies (sample)	Weighted mean (95% CI)	Weighted mean (95% CI)
Simoa**	6 (576)	23.3 (13.6–33.1)	0.22 (0.15–0.29)	6 (665)	5.22 (2.99–7.45)	0.06 (0.03–0.09)
Lilly (MSD)	7 (570)	24.4 (18.1–30.7)	0.54 (0.42–0.65)	7 (544)	8.06 (4.50–11.6)	0.18 (0.16–0.20)
Immunoprecipitation	2 (65)	224 (202–246)	0.44 (0.16–0.71)	2 (61)	50.2 (37.8–62.7)	0.11 (0.05–0.16)
WashU	1 (71)	10.5 (9.89–11.1)	2.72 (2.44–3.0)	1 (64)	2.90 (2.74–3.06)	0.75 (0.69–0.81)

Weighted means were obtained combining data from individual studies to perform meta-analyses of single-group continuous data

*CI* confidence interval

*CSF and plasma levels expressed as pg/mL

**Including the following: ALZpath, Janssen, Quanterix

About MSD, i.e., the most common laboratory technique (seven studies), pTau217 concentrations were highest in CSF (weighted mean [WM], 24.4; 95% CI, 18.1 to 30.7) and, to a lesser extent, in the blood (WM, 0.54; 95% CI, 0.42 to 0.65) in A+ subjects, while the A− group was characterized by sharply lower levels in both CSF (WM, 8.06; 95% CI, 4.50 to 11.6) and, particularly, blood (WM, 0.18; 95% CI, 0.16 to 0.20). Regarding Simoa, i.e., the second most widely used assessment method (six studies), the analysis highlighted a distribution overlapping with what was documented with MSD. Specifically, the A+ group showed higher levels in both CSF (WM, 23.3; 95% CI, 13.6 to 33.1) and blood (WM, 0.22; 95% CI, 0.15 to 0.29) as compared to A− participants who, similarly, displayed higher levels on the CSF (WM, 5.22; 95% CI, 2.99 to 7.45) than on blood (WM, 0.06; 95% CI, 0.03 to 0.09). Moreover, the only research that employed a mass spectrometry method reported a similar distribution of results, i.e., with the highest levels in A+ subjects, particularly in CSF (WM, 10.5; 95% CI, 9.89 to 11.1) and, to a lesser extent, in the blood (WM, 2.72; 95% CI, 2.44 to 3.0). In contrast, concentrations were lower in A− subjects and, again, higher in CSF (WM, 2.90; 95% CI, 2.74 to 3.06) than in blood (WM, 0.75; 95% CI, 0.69 to 0.81) [15]. Finally, one study evaluated an original IP-MS method in two different cohorts and documented a similar trend, i.e., the A+ group showed higher levels in the CSF (WM, 224; 95% CI, 202 to 246) as compared to blood (WM, 0.44; 95% CI, 0.16 to 0.71), whereas the A− participants were characterized by overall lower values than the previous group and, however, higher concentrations in the CSF (WM, 50.2; 95% CI, 37.8 to 62.7) than in blood (WM, 0.11; 95% CI, 0.05 to 0.16) [24]. In summary, all methods consistently demonstrated higher mean pTau217 levels in the CSF, representing the CNS compartment and, crucially, concentrations in the A+ group invariably exceeded those in the A− subjects, regardless of the biological substrate analyzed (i.e., CSF or blood). Notably, the IP-MS assessment method highlighted the highest CSF pTau217 levels in both A+ and A− groups [24]. At the same time, the mass spectrometry technique (i.e., WashU) showed the highest plasma pTau217 concentrations across both groups [15].

### Head-to-head meta-analysis

**Fig. 2:**
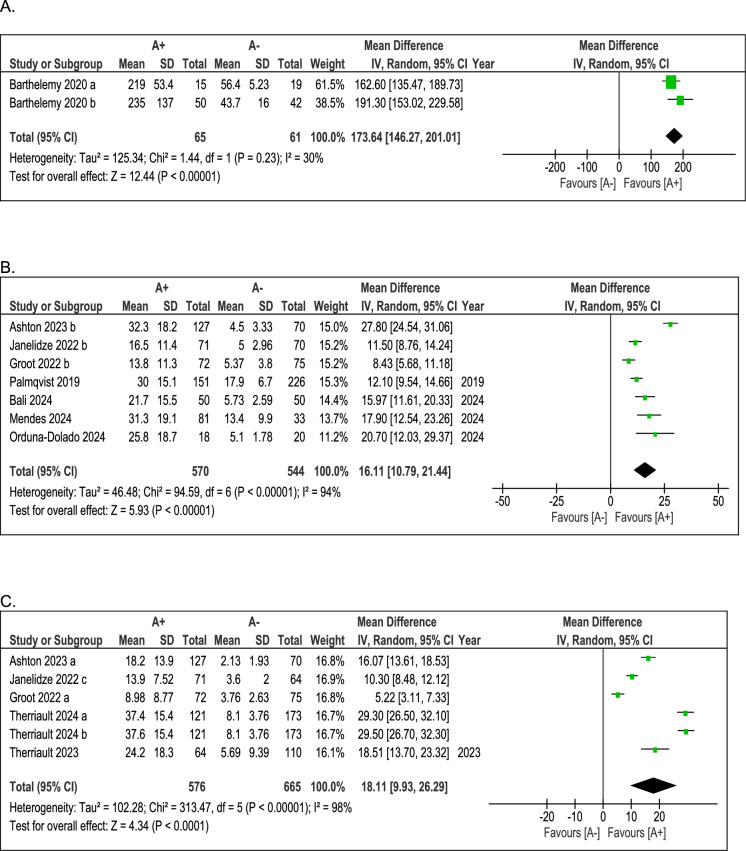
Results of the meta-analyses comparing the mean CSF levels (in pg/mL) among A+ versus A− subjects, stratified by assessment method (**A**: Immunoprecipitation; **B**: Lilly (MSD); **C**: Simoa). Abbreviations: *A+/−* Amyloid-positive/negative; *CSF*: cerebrospinal fluid; *MSD*: Meso Scale Discovery; *Simoa*: Single Molecule Array for Protein Detection

**Fig. 3:**
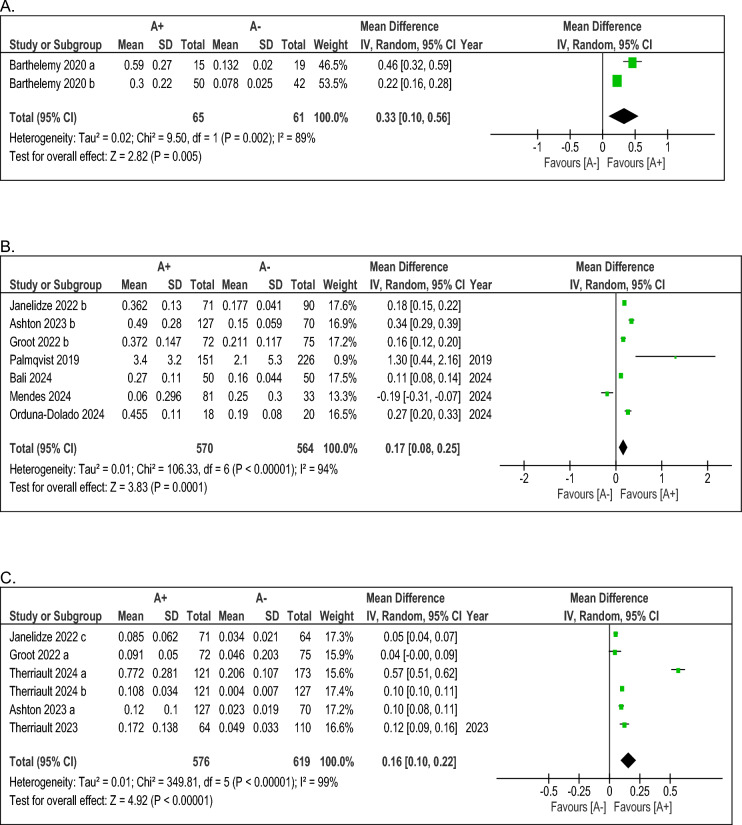
Results of the meta-analyses comparing the mean blood levels (in pg/mL) among A+ versus A− subjects, stratified by assessment method (**A**: Immunoprecipitation; **B**: Lilly (MSD); **C**: Simoa). Abbreviations: *A+/−* Amyloid-positive/negative; *CSF*: cerebrospinal fluid; *MSD*: Meso Scale Discovery; *Simoa*: Single Molecule Array for Protein Detection

While the previous meta-analysis provided a descriptive overview of mean pTau217 levels in CSF and blood within the A+ and A− groups, stratified by assessment method, this section will show a direct comparison of these measurements. The aim is to evaluate whether pTau217 concentrations can reliably distinguish between individuals in the two groups. Table 5 and Figs. 2 and 3 provide an overview of the results of the included studies in the head-to-head meta-analysis, stratified by assessment method (see Table 5 and Figs. 2 and 3).

**Table 5:** Results of the meta-analyses comparing the cerebrospinal fluid (CSF) and blood levels of pTau217 among Amyloid positive (A+) versus Amyloid-negative (A−) patients, stratified by assessment method (see also Fig. 2-3)

	N. studies (total sample)	N / N	Mean difference (95% CI)	p	I^2^, %
*1. pTau217 CSF levels (in pg/mL)*					
Immunoprecipitation	2 (126)	65/61	173.6 (146.3; 201.0)	<0.001	30
MSD	7 (1114)	570/544	16.1 (10.8; 21.4)	<0.001	97
Simoa*	6 (1241)	576/665	18.1 (9.93; 26.3)	<0.001	98
*2. pTau217 plasma levels (in pg/mL)*					
Immunoprecipitation	2 (126)	65/61	0.33 (0.10; 0.56)	0.005	89
MSD	7 (1134)	570/564	0.17 (0.08; 0.25)	<0.001	94
Simoa*	6 (1195)	576/619	0.16 (0.10; 0.22)	<0.001	99

*CI* confidence interval. *N/N* Total n. of subjects in the A+ group / Total n. of subjects in the A− group

*Including the following: ALZpath, Janssen, Quanterix

### CSF levels of pTau217 among A+ versus A− participants

Table 5 (first section) summarises the results about CSF pTau217 levels in A+ and A− groups (see Table 5). A direct comparison of pTau217 concentrations in CSF highlights that participants in the A+ group consistently exhibit, in a statistically significant way, higher values than those in the A− group, regardless of the assessment method employed. Specifically, about the MSD technique, mean pTau217 values were significantly higher in the A+ than in the A− group (mean difference [MD], 16.1; 95% CI, 10.8 to 21.4; *p* <0.001) (see Fig. 2, section B). Accordingly, Simoa also highlighted higher pTau levels in A+ subjects than in A− ones (MD, 18.1; 95% CI, 9.93 to 26.3; *p* <0.001) (see Fig. 2, section C). Finally, although assessed in only two patient cohorts, the IP-MS method also demonstrated its ability to differentiate between A+ and A− participants, yielding significantly higher values in the former compared to the latter (MD, 173.6; 95% CI, 146.3 to 201.0; *p* <0.001) (see Fig. 2, section A). Therefore, these findings underscore that evaluating pTau217 on CSF is a robust and reliable marker for distinguishing patients with amyloid pathology from those lacking this neuropathological characteristic.

### Blood levels of pTau217 among A+ versus A− participants

Table 5 (second section) provides an overview of the results of blood pTau217 values in the A+ and A− groups (see Table 5). Notably, the analysis showed results similar to those observed on CSF. Specifically, irrespective of the laboratory method employed, pTau217 assessments on blood demonstrated that they can reliably differentiate A+ from A− participants in a statistically significant way. In particular, the MSD technique showed that A+ participants are characterised by higher values than A− ones (MD, 0.17; 95% CI, 0.08 to 0.25; *p* <0.001) (see Fig. 3, section B), and the same was highlighted for the Simoa (MD, 0.16; 95% CI, 0.10 to 0.22; *p* <0.001) (see Fig. 3, section C). Finally, also on blood, IP-MS showed a statistically significant difference between A+ and A− subjects (MD, 0.33; 95% CI, 0.10 to 0.56; *p* = 0.005) (see Fig. 3, section A). Thus, crucially, blood pTau217 measurements have also demonstrated their ability to differentiate between A+ and A− individuals, reinforcing the distinction observed in CSF assessments and highlighting their potential as reliable tools for identifying amyloid pathology.

## Discussion

AD is the leading cause of dementia worldwide, and cost-effective tests to diagnose AD are a primary goal of global research [32, 33]. Here, to our knowledge for the first time, we show a comprehensive synthesis of available evidence enabling a direct comparison between pTau217 levels in CSF and blood. Two key findings emerge from our systematic review and meta-analysis: first, the mean pTau217 levels confirm a consistent gradient, being higher in CSF than in blood and, crucially, in A+ individuals than in A– ones, regardless of the laboratory method employed. Second, and most importantly, we highlight that all the considered laboratory techniques, i.e., MSD, Simoa, and IP-MS, can reliably distinguish A+ subjects from A– ones, whether applied to CSF or blood samples. Notably, previous research had already underscored that most individual studies assessing pTau217 in both CSF and blood reported a correlation, albeit of varying strength, between these measures [10]. This qualitative synthesis of the evidence, while insightful, did not allow quantitative evaluations of mean pTau217 levels or their utility in distinguishing between A+ and A– participants. By contrast, the present work addressed these limitations, demonstrating that blood pTau217, regardless of the laboratory technique employed, reliably differentiates A+ from A– individuals across the AD continuum. These findings hold substantial promise for both diagnostic and therapeutic advancements.

Indeed, it is well-established that AD symptoms arise even decades after the onset of the neuropathological alterations when significant brain damage has occurred and current therapies, while capable of slowing cognitive decline, cannot halt or reverse the neurodegenerative process [34]. Consistently, in recent years, research has increasingly focused on disease-modifying treatments, which are most effective in the early stages of AD, such as MCI, before the pathological protein burden becomes too severe [35]. Thus, the ability to detect amyloid pathology in its early stages is paramount and broadly implementable, safe, and non-invasive tools are urgently needed for identifying at-risk individuals most likely to benefit from these innovative therapies. In this context, blood-based biomarkers are emerging as a promising tool, offering a practical alternative to more invasive and less accessible methods such as lumbar punctures or PET scans. As they are more cost-effective and easier to obtain, they might represent accessible and scalable diagnostic tools. Indeed, the diagnosis of AD based on clinical criteria alone is challenging, also due to its atypical presentation, and the risk of misdiagnosis remains high both in specialised centers and, even more so, in primary care settings [36–38]. As a result, neuropathological biomarkers have become essential not only for confirming diagnoses but also for assessing eligibility for clinical trials [39–41]. Coherently, the use of biomarkers significantly reduces the rate of diagnostic errors [42]. Despite promising prospects, identifying suitable blood-based biomarkers for AD has proven challenging. Neurodegeneration markers, such as neurofilament light chains and total Tau protein, lack specificity and reflect unspecific neuronal damage from various conditions (e.g., stroke, multiple sclerosis), making them unsuitable to reliably identify AD-related pathology [42]. Therefore, research has focused on AD-specific biomarkers, particularly Aβ, a hallmark of AD neuropathology. While mass spectrometry-based plasma Aβ assays raised significant expectations, their diagnostic utility has been limited. Challenges include overlapping concentrations between Aβ PET-positive and PET-negative individuals and the significant peripheral production of Aβ [8]. Even newer immunoassay-based techniques face issues such as blood-related interferences and indirect Aβ detection [9].

In contrast, pTau has emerged as a specific AD biomarker. Specifically, abnormal Aβ accumulation triggers intracellular kinase activity, leading to Tau hyperphosphorylation, which disrupts its normal function, alters its structure, and promotes neurofibrillary tangle formation, underscoring its potential as a reliable indicator of AD pathology [43]. Consistently, blood-based biomarkers of pTau have shown strong correlations with AD pathology markers from CSF, PET imaging, and post-mortem analyses [5, 24, 44]. Moreover, plasma biomarkers could help identify marked Tau pathology, which is particularly relevant given the reduced efficacy of anti-amyloid therapies in advanced disease stages [45]. In addition, blood-based biomarkers of AD, especially in combination with indices of neurodegeneration, can accurately predict the subsequent development of dementia [46]. Importantly, as recently shown by our group, also blood-based pTau181 is a relevant biomarker for distinguishing A+ from A− individuals [47]. However, while pTau181 has historically been the most studied and established for its diagnostic role, emerging evidence suggests that other isoforms, particularly pTau217, show significant promise [10]. Consistently, some studies suggest that pTau217 outperforms pTau181. For instance, the former has demonstrated superior accuracy in identifying abnormal CSF and PET biomarker status and differentiating AD from other neurodegenerative diseases or controls compared to the latter [24, 48–50]. Importantly, the potential of pTau217 has been shown not only in CSF but also in blood, where it highlighted significant predictive power for identifying abnormal CSF Aβ status and progression from MCI to overt AD [25, 31]. Indeed, blood-based pTau217 proved high diagnostic performance for AD and strong correlations with amyloid and tau pathology [15, 50, 51]. Conversely, plasma pTau181 showed a certain degree of variability [48, 52]. Consistently, recent research highlighted that plasma pTau217 is more efficient than the isoforms 181 and 231 in identifying amyloid and tau alterations, as assessed by PET scans, and clinical phenotypes in a memory clinic cohort [27].

These findings are particularly relevant given that pTau181 alterations tend to occur at stages of AD when marked neuropathological changes are already present, whereas pTau217 appears to identify earlier stages of the pathological process [16]. This distinction is critical as it suggests that pTau217 may be useful in identifying individuals during presymptomatic stages of the disease or when only mild cognitive deficits are present—precisely the stages when disease-modifying therapies are likely to be most effective. Notably, evidence suggests that MCI patients, compared to those with overt dementia, exhibit the strongest correlation between blood and CSF biomarkers, likely because blood levels plateau in advanced neuropathological stages [53]. This makes MCI patients not only the ideal candidates for new drug trials but also the population in which blood-CSF biomarker alignment is most reliable [10]. Furthermore, recent single research documented not only that plasma concentrations of pTau217 follow a gradual increase from cognitively unaffected A− to A+ subjects but also that they reach ever-higher values in those who develop MCI and, above all, overt AD dementia [30]. These findings also align with research showing that plasma pTau concentrations correlate with age and, coherently, are lower in young people than cognitively normal elderly subjects [54]. Importantly, our meta-analysis not only confirms this trend (i.e., from A− to A+, single-group meta-analysis) but also underscores that blood pTau217, irrespective of the laboratory technique employed, can discriminate, in a consistently statistically significant way, subjects on the AD continuum from healthy controls or different disorders (head-to-head meta-analysis). This underscores its potential as a screening tool for reliably enrolling suitable patients in clinical trials during early disease stages. Although MSD and Simoa are currently the most widespread and used techniques, other methods seem to be very promising, especially considering that the IP-MS and the mass spectrometry alone showed the highest CSF and blood pTau217 levels, respectively, in both the A+ and A− groups [15, 24]. This suggests that mass spectrometry-based methods might detect even minimal changes in pTau217 levels, which will be crucial if cut-off values are available for early identification of A+ patients.

Recently proposed neuropathological diagnostic criteria for AD, although currently limited to research settings, recognise blood pTau alterations, including the pTau217 isoform, as core markers sufficient to establish a diagnosis [40]. These criteria also emphasise the high accuracy of pTau217 in mapping both the Aβ and AD tauopathy pathways. Crucially, all patients enrolled in this systematic review and meta-analysis were diagnosed with AD based on biomarker evidence. This provides strong confidence in our findings that blood-based pTau217 can accurately differentiate A+ from A− individuals, regardless of their cognitive status or disease stage. This ability may prove critical for its large-scale implementation. Indeed, as already suggested, this blood biomarker might be used as the first step in a screening process to identify patients for more specific, but also more costly and invasive, tests to increase the accuracy of diagnosis, which is common practice in many areas of medicine to increase the specificity of the screening test while limiting the use of unnecessary assessment tools [31, 32, 55]. Interestingly, a recently developed workflow demonstrated that utilizing a plasma pTau217-based model for risk stratification in patients with MCI can significantly reduce the need for confirmatory testing while accurately classifying patients, reserving more invasive assessments solely for uncertain cases [56]. Consistently, it has been shown that, in patients with symptomatic advanced dementia, there are marked concentration differences in plasma pTau217 levels, which are significantly elevated in AD patients. In contrast, they are normal in other neurodegenerative diseases [50]. This confirms that blood-based pTau217 represents a reliable signature of underlying AD neuropathology. Therefore, this biomarker can help reduce the need for invasive assessments in the differential diagnosis of AD, in monitoring disease progression, and in determining eligibility for clinical trials focusing on disease-modifying therapeutics [30]. In addition, recent evidence also suggests that blood pTau217 levels may also be relevant for assessing the efficacy of new disease-modifying therapies. Indeed, a recent clinical trial showed a significant reduction in plasma pTau217 levels following treatment with donanemab in patients with early symptomatic AD [57]. Taken together, evidence suggests relevant clinical applications for blood-based pTau217 in the AD continuum.

## Limitations

Despite conducting an extensive literature search and including a substantial cohort of 819 A+ and 1055 A– participants through rigorous selection criteria, our systematic review and meta-analysis acknowledge several limitations. First, the included studies applied highly heterogeneous criteria to categorize participants from biomarker perspectives. This variability introduces constraints on the generalizability of our findings. Moreover, the presence of neuropathological AD biomarkers does not necessarily predict clinical disease development, reflecting its multifaceted and complex nature [58]. In this context, introducing blood-based tests, ideally standardized, cost-effective, and reproducible across centers, might offer a promising pathway to simplify and enhance the categorization of individuals based on biomarker profiles [59]. Indeed, while clinical and neuropsychological assessments are relatively inexpensive and widely accessible, current neuropathological evaluations face challenges of invasiveness, cost, and accessibility [60]. The systematic adoption of blood-based biomarker evaluations could streamline diagnostic categorization, broaden participation in international clinical trials, and expand access to essential neurobiological classifications in resource-limited settings. Furthermore, although we stratified individuals as A+ or A–, future research could explore whether blood pTau217 levels reliably stratify individuals also based on cognitive status. Such an approach could complement biomarker-based classifications with clinical stratification, particularly as validated and standardized clinical tools become more widely available. It is also relevant to note that current research focuses only on blood or CSF pTau217 measurements due to economic, analytical, and invasiveness constraints [10]. This limits the ability to ascertain whether peripheral blood pTau reflects AD neuropathology in the CNS. Thus, the sample size of studies meeting the inclusion criteria for this systematic review was inherently limited. However, it still allowed robust statistical analyses demonstrating the ability of blood pTau to distinguish A+ from A– individuals. Future large-scale studies assessing pTau levels in both blood and CSF will be essential to confirm blood pTau as a reliable marker of the CSF status, potentially obviating the need for lumbar punctures or PET scans. It is hoped that this systematic review and meta-analysis will raise awareness of the need for both blood and CSF pTau217 measurements to address these issues. Another major limitation is the absence of standardized cut-off values for blood pTau217 to differentiate A+ from A– individuals. Indeed, these measures are strongly dependent on the specific methodology employed by each laboratory and, therefore, discrimination is only possible by direct comparison with (reasonably) healthy subjects [61]. Thus, multicenter longitudinal studies will be crucial to identify these cut-offs, which will be essential, especially in more complex cases, such as those with small fold-changes between patients and controls or amyloid PET positive and negative subjects [62]. Importantly, the mean values provided by this meta-analysis, based on a large sample size, might represent a starting point for establishing these critical cut-offs. A further limitation, unfortunately not assessable in this meta-analysis, as it was not explored in depth in the included studies, relates to BBB permeability, which affects blood pTau measurements and can be influenced by factors unrelated to AD pathology, such as age, hypertension, diabetes, and kidney disease [63, 64]. Although these comorbidities are likely similarly distributed across both A+ and A– groups due to the similar age of participants, their potential impact warrants caution in interpreting results [65]. Moreover, of note, some studies suggest that BBB permeability does not consistently influence blood pTau levels, hinting at roles for other pathways, such as interstitial fluid bulk flow, CSF absorption, or lysosomal degradation, which remain poorly understood and deserve further investigation [66]. Finally, our focus on pTau217 reflects its emerging recognition as a promising biomarker with significant diagnostic potential across the AD continuum. Indeed, recent evidence highlights that pTau217 demonstrates clinical performance comparable to or even superior to established CSF-based tests in detecting AD pathology, emphasizing its potential to revolutionize diagnostics and clinical stratification [67]. Thus, while other isoforms, such as pTau181, have been studied extensively, p-tau217 may offer distinct advantages by capturing earlier stages of AD neuropathology or reflecting specific pathological processes more sensitively [16]. Moreover, although other isoforms, such as blood pTau231, show promise in the earliest stages of the disease, the available evidence is still limited, and further studies are needed to confirm their potential [68]. Thus, future research should explore the utility of pTau217 across diverse stages of the disease, incorporating standardized methodologies to clarify its role relative to other isoforms. Such research will be instrumental in determining its diagnostic value and refining strategies for biomarker-based classifications in AD.

## Conclusions

In conclusion, our systematic review and meta-analysis confirm that blood-based pTau217 represents a promising biomarker for the AD continuum, with consistently higher levels in the CSF and, to a lesser extent, the blood of individuals with AD pathology, irrespective of the laboratory method used. Moreover, notably, blood pTau217 reliably differentiates A+ from A− individuals, regardless of cognitive status and laboratory method used, underscoring its potential for early diagnosis and inclusion in clinical trials. These findings advocate for integrating blood-based biomarkers into routine clinical practice for AD spectrum patients, reducing reliance on costly and invasive diagnostic tools. Importantly, this shift could also enhance diagnostic accessibility in resource-limited settings, addressing disparities in healthcare and fostering more equitable access to care worldwide.

## Supplementary Information

Below is the link to the electronic supplementary material.

Supplementary file1 (DOCX 31 kb)

## Data Availability

All data generated or analyzed during this study are included in this work and the articles in the bibliography. However, raw data (Excel spreadsheets) used in the statistical analyses will be made available upon reasonable request through the data analysis specialist (Prof. Maria Elena Flacco).

## Abbreviations

Aβ42: amyloid-β42
AD: Alzheimer’s disease
A+/-: Amyloid-positive/negative
BBB: blood-brain barrier
CNS: central nervous system
CSF: cerebrospinal fluid
IP-MS: immunoprecipitation with mass spectrometry
MCI: mild cognitive impairment
MD: mean difference
MSD: Meso scale discovery
PET: positron emission tomography
p-tau: phosphorylated Tau
Simoa: Single molecule array for protein detection
WM: weighted mean
